# Efficient simulations of mobility matrices for electrolytes by applying forces

**DOI:** 10.1039/d4sc03325f

**Published:** 2024-09-13

**Authors:** Pramudit Tripathi, Scott T. Milner

**Affiliations:** a Department of Chemical Engineering, The Pennsylvania State University University Park Pennsylvania 16802 USA stm9@psu.edu; b Department of Materials Science and Engineering, The Pennsylvania State University University Park Pennsylvania 16802 USA

## Abstract

Ion drift velocities in response to electric fields are a critical attribute of battery electrolytes. Accurately predicting species mobilities in such systems is an important challenge for atomistic simulations. In this work, we investigate two organic liquid electrolytes: LiPF_6_ dissolved in (a) dimethyl carbonate (DMC) and (b) a mixture of DMC and ethylene carbonate (EC). We compare two approaches to measure mobilities: observing center of mass diffusion with no forces applied, and observing species drift in response to external forces. The two approaches are related by the fluctuation–dissipation theorem, but they are not equally efficient computationally. We argue that statistical errors of the two methods scale differently with system size and simulation run time. In a head-to-head test, we apply both methods to LiPF_6_ in DMC in multiple simulations with the same size and run time. The drift method gives a much smaller variance in repeated measurements than the diffusion method, and should be preferred in practice.

## Abstract 2

Mobility matrices from drift measurements give more accurate conductivity than diffusion measurements.

## Graphical abstract

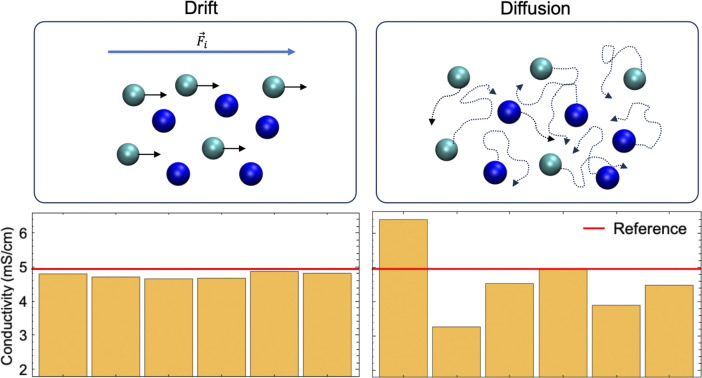

## Introduction

Electrolytes mediate ion transport between the electrodes in electric cells. A good electrolyte dissolves ions easily and readily allows solvated ions to move in response to an electric field, resulting in high conductivity and rapid charging and discharging of the battery. A good electrolyte should also exhibit a high transference number, *i.e.*, cations carry most of the current, which reduces concentration polarization during battery operation and increases the power density of the battery.^1^

Lithium-ion batteries have high energy density because of their high electrode potential and lightweight cations. However, lithium ions are small, and tend to stick to solvent molecules and counterions because of strong Coulomb interactions. These interactions result in ion clusters, which impede cation motion. Therefore, many experiments and simulations have been devised to study the interactions and mobility of ions and solvents in lithium-ion batteries.

Impedance spectroscopy has been widely used to characterize battery electrolytes. Alternating current is passed through the electrolyte, and the corresponding voltage drop and phase difference are measured over a range of frequency. The results are analyzed in terms of an equivalent circuit, which primarily provides information about the electrode/electrolyte interface, while treating the bulk electrolyte as a continuum.^2–5^ Impedance spectroscopy can measure conductivity;^6^ but high conductivity may arise from current carried by anions rather than cations.^7–10^

Experimental methods that characterize the motion of cations and anions separately are needed to provide a comprehensive picture of electrolyte transport. Pulsed-field gradient nuclear magnetic resonance^11^ can measure the self-diffusion coefficients of individual electrolyte species.^12–16^ However, self-diffusion alone only suffices to predict the mobility of electrolyte species in the dilute limit, in which the motions of anions and cations are uncorrelated.^17^ However, practical lithium-ion battery electrolytes are far from dilute.

More recently, pulsed-field gradient NMR has been applied to electrolytes in an electric field by an approach called electrophoretic nuclear magnetic resonance (ENMR), which can separately measure the drift velocities of cations, anions and solvents.^18^ Separate drift velocities of cations and anions suffice to determine the conductivity and the transference number.^17,19^ This method has been used in multiple studies on ionic liquid electrolytes containing lithium salts,^19–24^ solid polymer electrolytes^24–27^ and organic solvents.^28–30^

Overall, these experiments show that the motion of Li^+^ is strongly correlated with anions and solvent molecules. The correlated drift of species and their response to external forces are described by the linear response relation:1
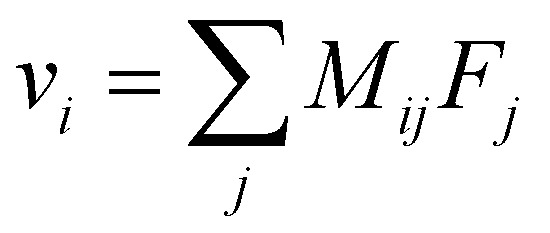
which, relates the drift velocity *v*_*i*_ of species *i* to the force *F*_*j*_ applied to species *j*. The mobility matrix *M*_*ij*_ is symmetric and positive definite. The off-diagonal terms of *M*_*ij*_ describe the correlated motion of different species: a force on one species can result in the drift of another.^31^

As a consequence of the fluctuation–dissipation theorem, the mobility matrix *M*_*ij*_ also describes the correlated diffusion of species with no forces applied:2
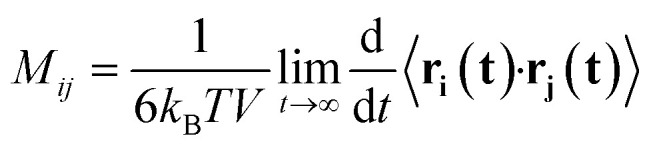
where **r**_**i**_(**t**) is the displacement of the center of mass of *i*^th^ species with respect to the center of mass of the system at time *t*, *k*_B_*T* is the thermal energy and *V* is the system volume. Intuitively, drift in response to applied forces proceeds by biased diffusion. The more readily a species diffuses, the faster it drifts in response to a force. Likewise, if two species tend to diffuse together, a force on one will cause the other to drift as well.

In recent years, Fong *et al.* have characterized polymerized ionic liquid electrolytes using Onsager transport coefficients obtained from correlated diffusion of species.^32–34^ Recently, Fang *et al.* adapted this formulation for binary solvents evaluating transport properties with respect to the center of mass frame of both solvents.^30^

In simulations, nonequilibrium methods with external forces have been used to evaluate transport properties.^35,36^ The theoretical basis for these simulations was formalized through linear and nonlinear response theory.^35,37–39^ These simulation algorithms were used to evaluate transport of gases in zeolite pores.^40,41^ Subsequently, Wheeler and Newman adapted a similar approach for salt solutions.^42^

Thus there are two ways to measure mobility matrices: observing drift of species in response to forces, or observing the diffusion of the center of mass of species. Both methods incur statistical error, but is one approach more efficient than the other? This question has received scant attention in previous work.

Recently, Shen *et al.* concluded that measuring conductivity by observing ion drift under an electric field is more efficient and accurate than characterizing the mobility matrix using the diffusion approach.^43^ However, observing drift under an electric field does not determine the entire mobility matrix, therefore the two measurements by Shen *et al.* are not equivalent to each other.

In this work, we compare the two methods of measuring mobility matrices. Lithium-ion battery electrolytes are ideal for this comparison because they are concentrated multi-component mixtures with substantial interactions between different species. We chose two well-studied liquid electrolytes: lithium hexafluorophosphate (LiPF_6_) dissolved in dimethyl carbonate (DMC), and LiPF_6_ dissolved in equal parts by weight of dimethyl carbonate and ethylene carbonate (DMC/EC).

For simulations to reflect experimental reality, interaction potential must be realistic. Because lithium ions stick to anions and polar moieties, tuning and validating these interactions is essential. We tune and validate the non-bonded force field parameters of LiPF_6_ in DMC by matching the experimental osmotic pressure *versus* salt concentration to simulation results.^44^ The strength of lithium ion attraction to anions and solvent strongly affects ion clustering, which strongly affects the osmotic pressure. We study LiPF_6_ solvation and perform cluster analysis in both systems.

We compare the computational efficiency of the two methods of measuring mobility matrices in two ways. First, we construct scaling arguments for both methods, predicting how statistical error depends on system size and simulation run time. Then, we directly compare the errors in practice in terms of conductivity and transference numbers, by applying both methods to multiple instances of the same system with the same total run time.

## Calibrating force fields with osmotic pressure

Force field parameters that accurately represent the intermolecular interactions are crucial for getting realistic ion transport predictions. Force field parameters are usually validated by comparing simulated properties to experimental results for boiling point, vapor pressure and density. For electrolytes, interactions of cations with anions and polar solvents are particularly important, so we choose osmotic pressure of LiPF_6_ dissolved in dimethyl carbonate (DMC) at a range of concentrations to validate the force field parameters.

Osmotic pressure is well suited for calibrating force field parameters in liquid electrolytes when available, because it depends sensitively on the size of ion clusters, which also affects the number of available charge carriers and hence the conductivity. When ions cluster, the osmotic pressure drops, because the ions no longer move independently to explore the solution volume. We measure osmotic pressure over a range of concentrations in a single molecular dynamics simulation using a method developed in our group,^44^ and compare it with the experimental osmotic equation of state for LiPF_6_ dissolved in DMC^45^ as a check on our non-bonded force field parameters.

**Fig. 1:**
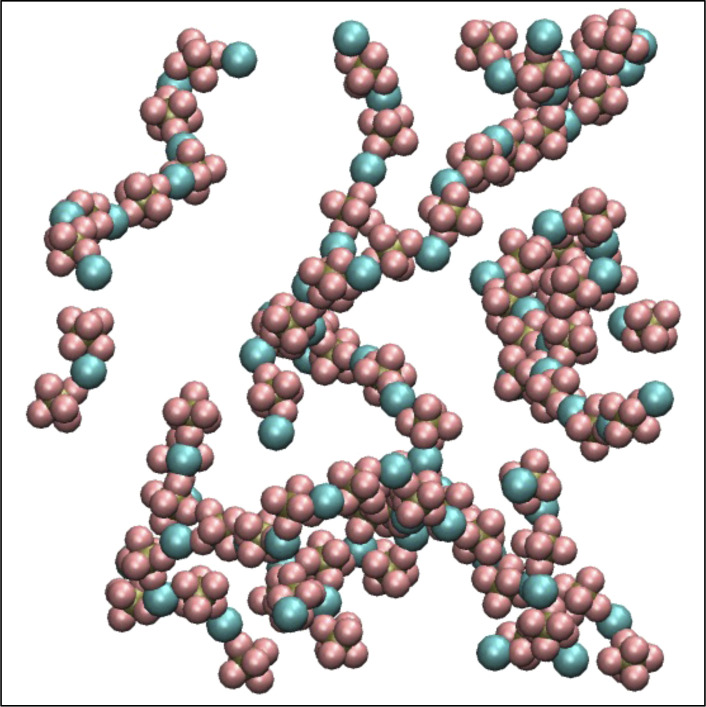
Li^+^ and PF_6_^−^ ions form long ion strings when equilibrated with non-bonded force field parameters from Kumar *et al.*^46^

**Fig. 2:**
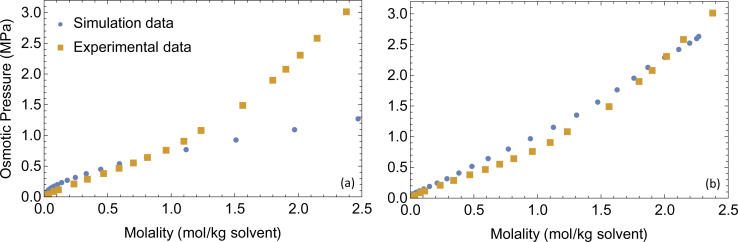
Simulated and experimental osmotic pressures (a) before and (b) after optimizing the force field parameters.

A set of force field parameters for LiPF_6_ solvated in ethylene-carbonate (EC) were obtained by Kumar *et al.* with partial charges obtained from density functional theory by electrostatic fitting.^46^ Because molecular structures of DMC and EC are similar, we adapted the Lennard-Jones parameters from EC for DMC, and obtained partial charges for DMC by electrostatic fitting (see Table 1). However, equilibration of LiPF_6_ dissolved in DMC using these partial charges gives long string-like ion aggregates (see Fig. 1), and a simulated osmotic pressure much smaller than experiments (see Fig. 2a). The long ion strings and low osmotic pressure result from poor solvation with these non-bonded potential parameters.

**Table 1:** Optimized Lennard-Jones force field parameters (*σ*, *ε*) and partial charges (*q*) used for LiPF_6_ in DMC. Changes made over Kumar *et al.*^46^ are shown in boldface type

Atom/pair	*σ* (nm)	*ε* (kJ mol^−1^)	*q*(*e*)
O_*x*_	0.296	0.87864	**−0.430**
C_*x*_	0.375	0.43932	**0.590**
O_*s*_	0.300	0.71128	**−0.330**
C	0.350	0.27614	**0.160**
H	0.250	0.12552	**0.030**
Li	0.14424	0.43154	1.000
P	0.36950	0.55099	1.070
F	0.29347	0.12015	−0.345
Li–O_*x*_	**0.213**	**0.51314**	
Li–P	0.3007	0.05871	
Li–O_*s*_	0.20217	0.87600	
F–H	0.23951	0.86023	
F–C_*x*_	0.29381	0.27388	

To improve the agreement of simulations with the experimental osmotic pressure, the ions need to form smaller clusters, which can be achieved through better solvation of the ions. To achieve this, we reduce the short-range repulsive Lennard-Jones interactions between the Li^+^ ions and the carbonyl oxygen of the solvent molecules (O_*x*_), to which the cations are most strongly attracted, following the method described by Gillespie *et al.*^44^ We change both *σ* and *ε* such that the only the repulsion factor (4*εσ*^12^) reduces, keeping the attraction factor (−4*εσ*^6^) unchanged. This reduction in repulsion makes the Li^+^ more likely to associate with the solvent molecules instead of clustering with anions.

Additionally, lithium ions polarize neighboring molecules, which causes dielectric screening of Coulomb interactions.^47^ To account for this dielectric screening, charges are often scaled by a factor ranging from 0.75 to 0.85 in molecular simulations containing lithium ions.^47–49^ Instead of this *ad hoc* charge scaling for ions, we use a background dielectric constant of *ε* = 2, which corresponds to scaling by a factor of 1/2 ≈ 0.71 all partial charges (not just ions). This approach is physically motivated, insofar as Coulomb interactions between all charges would be screened by the electronic polarizability of the liquid, which typically contributes a dielectric constant of about *ε* = 2 for organic liquids.

The combination of these two changes improves ion solvation in simulations; as a result, we achieve good agreement with the experimental osmotic pressure (see Fig. 2b). The final non-bonded Lennard-Jones parameters and partial charges use the OPLS-AA force field and are given in Table 1 with changes made from Kumar *et al.* parameters in boldface type.

## Lithium solvation

Having settled on force field parameters, we begin by exploring the equilibrated structure of solvated ions. We examine two different systems relevant to the contemporary lithium-ion batteries with organic liquid electrolytes: (a) 0.75 M LiPF_6_ dissolved in dimethyl carbonate (DMC) and (b) 0.75 M LiPF_6_ dissolved in equal parts by mass of dimethyl carbonate (DMC) and ethylene carbonate (EC).

We construct the equilibrated solutions as follows: all molecules were initially inserted randomly into an 8 nm cube with periodic boundary conditions. After energy minimization, the solution was relaxed for 4 ns in an NVT simulation followed by 10 ns of NPT simulation. After achieving a constant equilibrium density, the solution was simulated under NVT conditions for 500 ns at 300 K to give the ions time to diffuse across the simulation box and form equilibrated clusters. Cluster analysis was performed on the final 200 ns of the simulation trajectory.

To identify ion clusters, we define a cutoff radius *R*, below which a Li^+^ cation is considered to be in close contact with PF_6_^−^ anion. The cutoff radius *R* is taken from the radial distribution function of Li^+^ with phosphorous atom of PF_6_^−^ as the location of the first minimum, *R* = 0.4 nm.

**Fig. 3:**
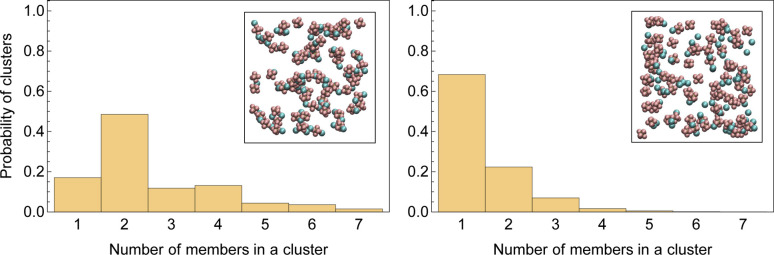
Histograms of sizes of randomly picked clusters in LiPF_6_ in DMC (left) and LiPF_6_ in DMC/EC (right). The inlaid images show an arbitrary snapshot of ion clusters.

Fig. 3 displays a histogram of cluster size for both systems. Clusters with even numbers of ions are evidently neutral. In the first system, almost half of the clusters are neutral pairs, and their motion does not directly contribute to the conductivity of the electrolyte. A small fraction of ions form single dissociated ions, and a large number of ion clusters have even more than two ions. In contrast, in the second system, a majority of ions are present as dissociated single ions and only a small fraction of the clusters are neutral pairs.

**Fig. 4:**
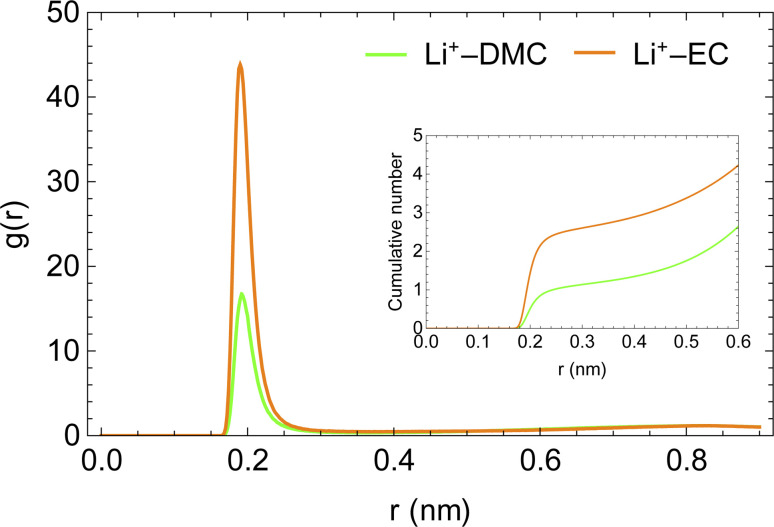
Radial distribution functions of carboxyl oxygens of solvent molecules with respect to Li^+^ ion in LiPF_6_ in DMC/EC system.

The enhanced availability of charge carriers in the presence of EC in the solvent mixture reflects the excellent solvation of lithium ions in EC. EC has a large dipole moment compared to DMC, which helps solvate ions like lithium efficiently.^50^Fig. 4 presents the radial distribution functions of the carboxyl oxygen in solvent molecules (DMC and EC) in the mixed solvent system with respect to Li^+^. A lithium ion is approximately three times more likely to be near EC than near DMC. As the solvent mixture is nearly equimolar, the affinity of Li^+^ for EC is evidently much stronger than DMC.

## Mobility in electrolytes

The linear response formulation of transport properties assumes a reference frame in which the center of mass (COM) is at rest. As we intend to study the system under external forces, we adopt the following convention: for any explicit force applied to species *i*, an implicit equal and opposite force is applied to the whole system. Thus the total applied force will always be zero and the COM of the system will remain at rest. The implicit force is distributed over the species proportional to their mass fraction *ρ̂*; thus, it has no effect on the drift of species with respect to the COM. Therefore, the mobility matrix must annihilate the mass fraction vector:^31^

3 **M**·**ρ̂** = **0**

If two species interact in a mixture, they exert intermolecular forces on each other. Consequently, motion of one of the species affects the motion of other species. These interactions are expressed by the off-diagonal elements of the mobility matrix. For example, the element *M*_12_ expresses the drift velocity of the first species when a unit force per volume is applied to the second species and all other explicit forces are zero. Onsager reciprocity dictates that the effect of species *i* on *j* equals the effect of species *j* on *i*, expressed as *M*_*ij*_ = *M*_*ji*_.

We demonstrate the linear response measurement of mobility matrices and prediction of transport properties for electrolytes using the two previously equilibrated systems: LiPF_6_ dissolved in DMC and LiPF_6_ dissolved in equal parts by weight of DMC/EC. The first system has three species: cations, anions and solvent molecules; the second has four species, with two types of solvent molecules.

In simulations, we can apply constant forces to any species we choose. The drift velocities in response to these forces can be measured to determine the mobility matrix. We apply a force on the COM of one species (and an equal and opposite force on the system COM) and measure the drift velocity of the species in the direction of the applied force.

The applied forces must be small enough to be in the linear response regime, *i.e.*, the drift velocities should be proportional to the force. We verify this by applying forces of the order of *kT*/Å per particle on one of the species and measuring the resulting drift velocities. We find that forces of 0.25*kT*/Å per particle lie comfortably in the linear response regime. This magnitude is used in all further simulations with applied forces.

For a system with *n* components, *n* simulations suffice to determine all the elements of the mobility matrix. For a three-component system we perform three simulations, in which forces are applied to the cations, anions, and solvent. For example, when we apply an explicit force *f*_11_ on the cations with implicit equal and opposite force on the system COM, we measure the drift velocities *v*_*i*1_ for species *i*. Combining the results of these three simulations, we have4
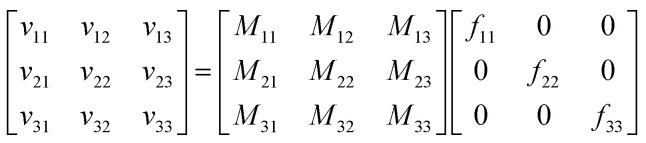
which can be solved for **M** as

5 **M** = **v**·**F**^−**1**^

Actually, *n* − 1 simulations are enough if we enforce the requirement that mobility matrix must be orthogonal to the mass fraction vector (see eqn (3)). In this work, we choose to run *n* simulations to treat all species equally, and obtain a check on our results by verifying that **M** satisfies eqn (3).

**Fig. 5:**
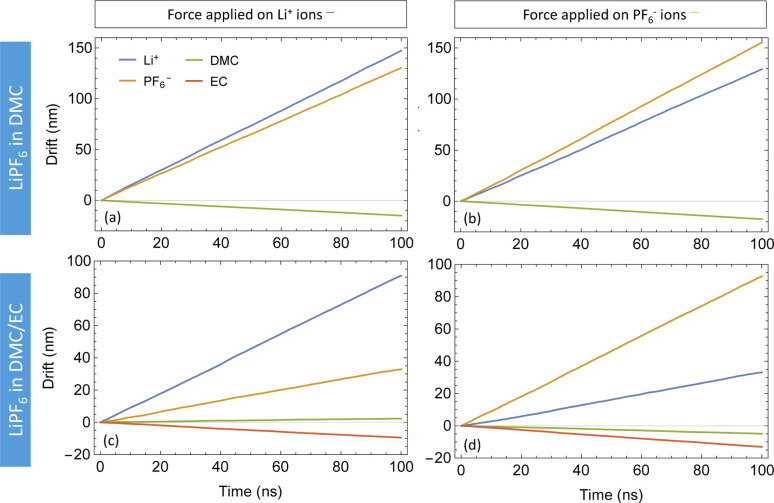
Drift plots of species in LiPF_6_ in DMC (top) and LiPF_6_ in DMC/EC (bottom) with forces on Li^+^ (left) and PF_6_^−^ (right).

Fig. 5 depicts the drift *versus* time of species obtained by applying forces (*f*_*jj*_ = 0.25*kT*/Å per particle) to both DMC and DMC/EC systems. With only DMC as a solvent for LiPF_6_, the cations and anions follow each other very closely. With a force on only one ion species, its counterions also move in the same direction at nearly the same drift velocity (see Fig. 5(a) and (b)). This observation confirms strongly coupled motion of Li^+^ and PF_6_^−^ in DMC; moving ions drag the counterions with them. In the DMC/EC mixture, the motion of ions is less correlated; ions still follow each other but not as closely as in DMC. Additionally, ions in DMC/EC drift a lot slower than in DMC.

The measured drift velocities with forces applied to each species in the electrolytes were used to calculate the mobility matrices for both systems. **M**_**1**_ and **M**_**2**_ correspond respectively to the pure DMC and mixed DMC/EC systems:6
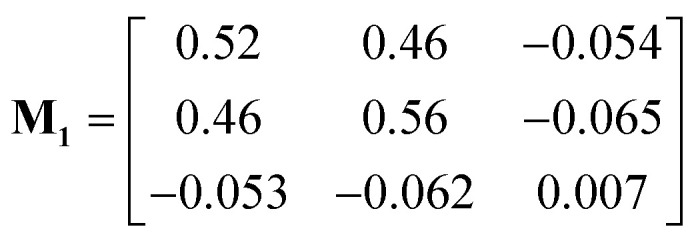
7
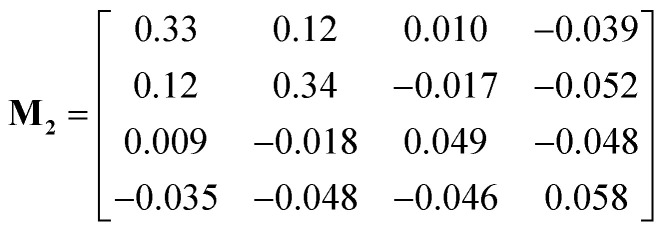


The units of the mobility matrices are of nm ns^−1^ per kJ mol^−1^ nm^−1^ nm^−3^, which is just velocity over force per unit volume in Gromacs units.

Both **M**_**1**_ and **M**_**2**_ are symmetric within computational error, following Onsager symmetry. In our chosen convention, the first and second rows and columns represent the cations and anions; the third row and column represent the solvent in **M**_**1**_. For **M**_**2**_, the third and fourth rows and columns correspond to EC and DMC. Note that some of the off-diagonal elements of **M**_**1**_ and **M**_**2**_ are negative, which means the corresponding species are anti-correlated in their drift. The solvent moves opposite to the explicit force on the ions because of the implicit force, which keeps the system COM stationary.

The first system (**M**_**1**_) has higher mobility values than the second (**M**_**2**_), because the ions stick more to each other and less to DMC. For the ions, the diagonal (*M*_11_ and *M*_22_) and off-diagonal (*M*_12_) values are very close, which corresponds to strongly correlated motion of cations and anions. In contrast, the ions in mixed DMC/EC have lower mobility, because cations stick more strongly to EC. Likewise, the off-diagonal ion mobilities in **M**_**2**_ are much lower than ion diagonal terms, because the motion of cations and anions are not so correlated.

The ionic conductivity (*κ*) and transference number (*t*_0_^+^) for an electrolyte can be measured by ENMR. We calculate *κ* and *t*_0_^+^ by two methods. First, we apply a constant electric field, in which the ions experience equal and opposite forces and drift in opposite directions. The resulting drift velocities can be used to calculate *κ* and *t*_0_^+^ using the following relations:8
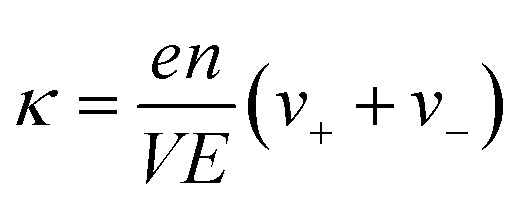
9
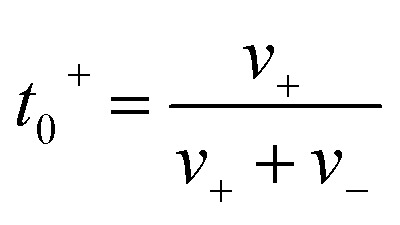
where *v*_+_ and *v*_−_ are the magnitudes of cation and anion drift velocities with respect to the system COM, 
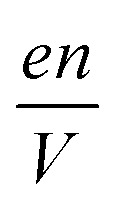
 is the charge density of the system, and *E* is the electric field strength.

Alternatively, we take the dot product of the mobility matrix of the electrolyte with the force vector representing an electric field to get ion velocities. The forces per unit volume resulting from a field *E* acting on the electrolyte are given by **F**_**E**_:10
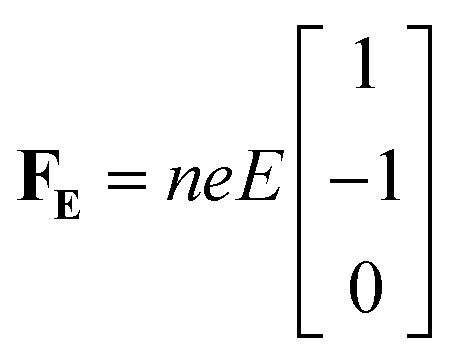
where *n* is the number of salt molecules dissolved in the electrolyte and *e* is the electronic charge. The elements of this vector represent the forces on cations, anions, and solvent respectively. The dot product **M·F**_**E**_ gives the drift velocities, which can be used to calculate conductivity (*κ*_pred_) and transference number (*t*_0,pred_^+^) from eqn (8) and (9).

Table 2 presents results for conductivity and transference number for both systems, obtained from *M* and directly from simulations in an electric field. The close agreement between the two approaches confirms that simulated transport properties of electrolytes in electric field are well predicted by our mobility matrix results.

**Table 2:** Ionic conductivity (*κ*) and transference number (*t*_0_^+^) for both systems measured directly by applying electric field (direct) and predicted by mobility matrix (pred). Experimentally measured conductivity (expt) is also included for reference

Property	System 1	System 2
*κ*_direct_ (mS cm^−1^)	4.97	12.64
*κ*_pred_ (mS cm^−1^)	4.74	12.72
*κ*_expt_ (mS cm^−1^)	5.16 (ref. 51)	12.08 (ref. 52)
*t*_0,direct_^+^	0.41	0.49
*t*_0,pred_^+^	0.38	0.49

Experimental measurement of transference numbers is more involved than conductivity. Researchers have used many methods to determine transference numbers of electrolytes, which often depend on questionable assumptions, and yield varying results.^53^ To avoid these complications, instead of comparing simulated transference numbers with experiments, we choose to compare self-diffusion coefficients of the ions in a 1 M solution of LiPF_6_ in DMC/EC at 300 K. Self-diffusion coefficients of Li^+^ and PF_6_^−^ from simulations are 3.64 × 10^−10^ m^2^ s^−1^ and 3.62 × 10^−10^ m^2^ s^−1^ respectively. The corresponding experimental values are 2.52 × 10^−10^ m^2^ s^−1^ and 3.86 × 10^−10^ m^2^ s^−1^.^54^

## Comparing methods to measure *M*_*ij*_

Because of the fluctuation–dissipation theorem and the resulting Green–Kubo relation, the mobility matrix *M*_*ij*_ can be obtained from simulations in two ways: (a) by applying a force *F*_*i*_ to species *i* and measuring the drift velocity *v*_*j*_ of species *j*, and (b) by measuring the time correlation between the COM displacements of species *i* and *j* as they diffuse in the absence of applied forces. Both approaches determine *M*_*ij*_ from averaged quantities (the average drift velocity, and the time correlation of COM displacements), and both averages necessarily incur statistical errors. The question arises: given finite computational resources, which approach is more efficient in terms of statistical error for a given total computational time? Although these approaches are formally equivalent by the fluctuation–dissipation theorem, it turns out they are not equivalent in terms of efficiency.

First, we consider the fluctuation approach (method B). The COM displacement **r**_*j*_(*t*) of species *j* executes a Gaussian random walk, with some effective step size *a* and step time *τ*. Irrespective of the system size, **r**_*j*_(*t*) is a single random walk; as a result, the average 〈**r**_*i*_(*t*)·**r**_*j*_(*t*)〉 benefits only from time averaging and not from a large system size.

**Fig. 6:**
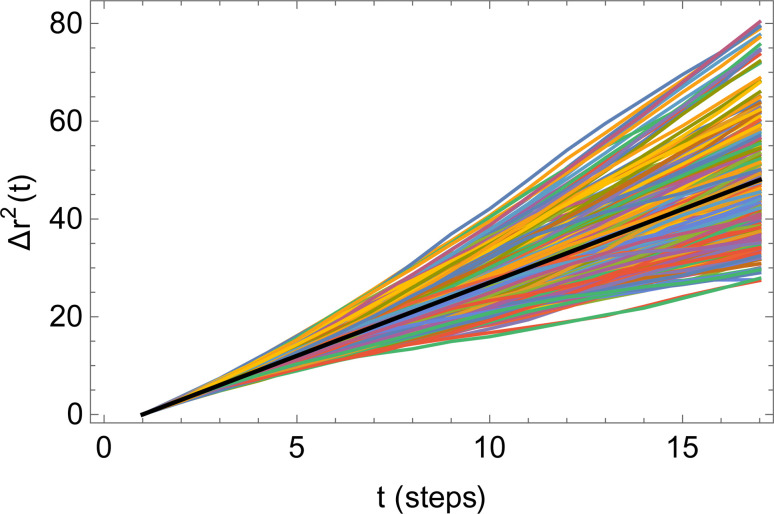
Time-averaged square displacements (〈**r**_**i**_^2^(**t**)〉) for 256 different random walks, each of 128 steps (colored curves), compared to the true mean-square displacement (black line).

Fig. 6 illustrates the behavior of the time-averaged mean-square displacement of 256 instances of random walks, each consisting of 128 Gaussian-distributed steps of unit mean-square length (colored curves), compared to the true mean-square displacement (black), which is given by:

11 〈**r**_**i**_^2^(**t**)〉 = 6*Dt*

with *D* = 1/2 for a random walk with Gaussian distributed steps of unit variance (see Appendix). The individual curves deviate smoothly from the true result, with an error that grows faster than linearly with the number of steps. As a result, taking the slope of an individual curve to measure the corresponding coefficient in the mobility matrix is plagued with statistical error. In fact, the relative error *E*_*R*^2^_ in the mean-square displacement grows with number of steps, according to eqn (12):12
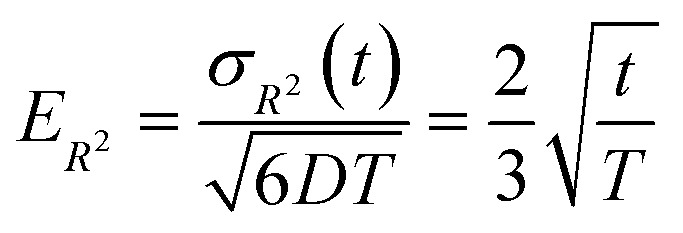
where *σ*_*R*^2^_(*t*) is the root mean squared error in the mean-square displacement, and *T* the total number of steps in the random walk. A detailed explanation for this result is presented in the Appendix. If *k* different walks are averaged, the relative error is reduced by a factor *k*^−1/2^, which is same as the error in a single random walk of *Tk* total steps used to evaluate the correlation function. In terms of total computational time, there is no benefit to averaging over multiple runs as opposed to using a single long run.

Therefore, for the correlation method, it is recommended to use the smallest practical time delay *t* to define the diffusive slope, and to use the smallest practical system size, since there is no benefit from averaging over different molecules. However, the time delay must be greater than the velocity autocorrelation time *τ*, below which the COM displacements do not behave as Gaussian random walks. Likewise, the system must be sufficiently larger than the correlation length of the solution, below which the simulation does not behave like a representative sample of a large system. Correspondingly, there must be a minimum number of particles N* ≫ 1 in any reasonable simulation.

In contrast, measuring the drift velocity *v*_*i*_ (method A) benefits from averaging over the molecules of component *i* as well as averaging over time. The statistical error in the drift velocity arises from simultaneous diffusive motion of the molecules as they drift along in response to the applied force. In measuring the drift velocity, we typically apply the largest force within the limits of linear response. On physical grounds, linear response should begin to fail when the drift velocity is of order *a*/*τ*, where *τ* is the velocity autocorrelation time and *a* is the diffusive mean-squared displacement of a molecule on the timescale *τ*. The relative error *E*_D_ in the total drift displacement of the set of *N* molecules then scales as:13
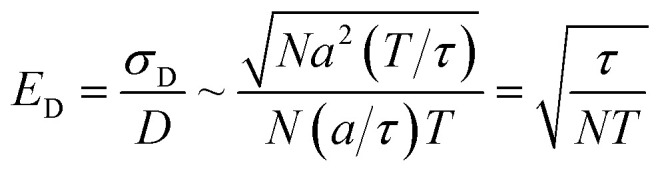


We can now compare the relative errors *E*_*R*^2^_ and *E*_D_ for equivalent computational resources *C*. In molecular dynamics simulations, the computational resources scale linearly with the number of particles *N* and the computational time *T*. So we can replace *NT* = *C* in the eqn (12) and (13), arriving at the final result:14
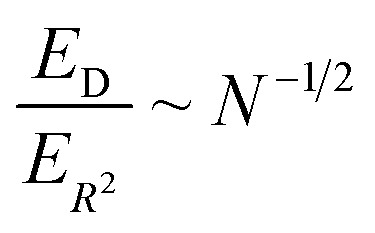


The relative error in measuring the drift velocity is smaller than the error in COM mean-squared displacements by a factor of the square root of the number of molecules *N*, because the drift velocity measurement benefits from averaging over molecules whereas the COM diffusion measurement does not.

To conduct a fair comparison between method A and B, we calculate the mobility matrices by both methods on the same equilibrated system of 0.75 M LiPF_6_ in DMC, containing 240 ions of each type at 300 K. We ran six replicates of 120 ns simulations for each method using the same computational resources to calculate the mobility matrices, which were used to calculate the key transport properties of the electrolyte: conductivity (*κ*) and transference number (*t*_0_^+^). Table 3 presents the results along with the standard errors. The transport properties are compared against reference values derived from simulations with an electric field.

**Table 3:** Ionic conductivity (*κ*) and transference number (*t*_0_^+^) values predicted by mobility matrices calculated using methods A and B. These predicted properties are compared to values measured by applying electric field to the system (reference)

Property	Reference	Method A	Method B
*κ* (mS cm^−1^)	4.97 ± 0.05	4.76 ± 0.03	3.94 ± 0.43
*t*_0_^+^	0.41 ± 0.01	0.42 ± 0.03	0.56 ± 0.07

**Fig. 7:**
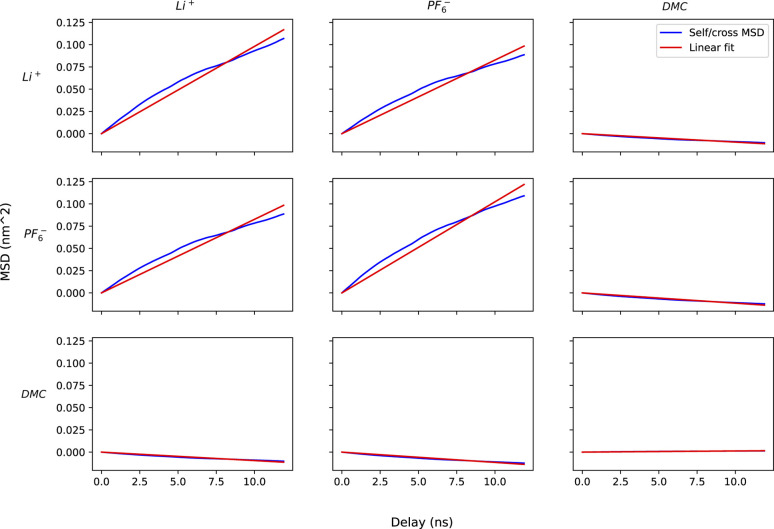
Grid of plots of mean squared displacements of center of mass of species (blue) and their corresponding linear fits to extract slopes. The on-diagonal and off-diagonal plots represent self and cross mean-squared displacement *vs.* time delay respectively.

For method A, we run six sets of drift simulations as described in the previous section, with forces applied for 40 ns each to cations, anions and solvent, totaling to 120 ns per measurement. For method B, we run six 120 ns simulations in which ions and solvent diffuse freely. We extract the COM trajectories of each species, and calculate self and cross mean-squared displacements up to a time delay of one-tenth of the simulation time (12 ns). Increasing time delay beyond a small fraction of the simulation will increase the error drastically as justified by the scaling argument (see Appendix). Fig. 7 shows the self and cross mean-squared displacement curves (blue) and best linear fits passing through the origin (red). The fitted slopes are used to calculate the mobility matrices, using eqn (2).

**Fig. 8:**
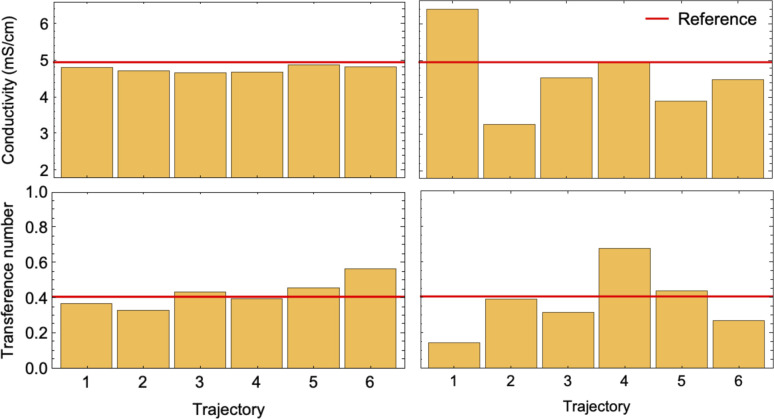
Bar charts of conductivity (top) and transference number (bottom) calculated using method A (left) and method B (right) for 6 different 120 ns simulations. Red horizontal lines show the reference values.

Fig. 7 confirms the correlated motion of Li^+^ and PF_6_^−^ ions. The motions of ions and solvent are anti-correlated, as expected from the results of method A. However, the mean-square displacement curves are not beautifully linear; much longer simulations are needed to obtain accurate results using method B. As evident from Table 3, method B incurs larger standard error in key transport properties than method A. Correspondingly, the average *κ* and *t*_0_^+^ values calculated from method A lie much closer to the reference values obtained from simulations with electric fields. Fig. 8 shows the run-to-run variation in *κ* and *t*_0_^+^ among the six replicates calculated by both methods. The narrow variance achieved using method A, together with the close agreement of transport properties with the reference values, establish it as a more reliable means of characterizing mobility properties in simulations.

## Conclusions

To conduct realistic atomistic simulations, we calibrate LiPF_6_ and dimethyl carbonate force field parameters by comparing experimental osmotic pressure with simulations. We also simulate our electrolytes with a background dielectric constant of *ε* = 2 to account for dielectric screening of Coulomb interactions from local polarization effects of Li^+^ ions in the solvent. We further conclude this optimized set of force field parameters produce realistic simulations to predict dynamic properties of the chosen lithium-ion electrolytes, based on agreement with the experimental self-diffusion coefficients of their constituent species and their ionic conductivity.

We study two lithium-ion electrolytes: LiPF_6_ in DMC, and LiPF_6_ in DMC/EC (1 : 1 by weight), evaluating their mobility matrices by applying forces to electrolyte species and measuring their drifts. The conductivity and transference numbers predicted by the mobility matrices agree with experimental studies and values measured by applying electric fields in the simulations within the computational errors.

Finally, we compare the two methods of evaluating mobility matrices: (a) by measuring the drift of electrolyte species in response to forces, and (b) by calculating mean-squared displacements of the COM of the electrolyte species, by simulating LiPF_6_ in DMC. We present a scaling argument for the statistical error, demonstrating that the drift method benefits from a larger number of molecules in the simulation whereas the COM diffusion method does not, supporting the efficiency of the drift method over the diffusion method. This argument is strengthened by much lower variances in six independent replicates of conductivity and transference numbers calculated by the drift method compared to the COM diffusion method.

## Appendix

The mean-square displacement Δ*r*^2^(*t*) computed for each of an ensemble of random walks (see Fig. 6) exhibits a striking behavior: the variation in the value at a given delay *t* grows faster than the average; the “sheaf” of colored curves “fans out” from the mean value (black line).

Because the relative error grows with the time delay *t*, measures of the slope of the mean-square displacement Δ*r*^2^(*t*) computed from a single random walk become increasingly unreliable for long time delays. In this Appendix, we provide a scaling argument that explains this behavior, and an analytical calculation of the leading dependence with prefactor of the relative error *versus* delay time.

Qualitatively, the increasing error at long delay times *t* arises because a random walk of a given length consists of progressively fewer independent sequences of length *t*. We may regard the average over a single random walk of Δ*r*^2^(*t*) as being performed by moving along the time axis, and computing the square displacement for each starting time *t*_0_. But successive contributions to the average are correlated, and thus do not improve the statistical error of the average, until we have moved along the time axis to an uncorrelated portion of the walk, a time *t* later.

For a walk of total length *T*, we have *S* = *T*/*t* independent random walk segments. The mean-square displacement Δ*r*^2^(*t*) is effectively an average over *S* independent experiments. The error bar for Δ*r*^2^(*t*) for a single random walk of length *t* is as big as the average itself, which scales with *t*. Averaging together *S* uncorrelated instances reduces the error bar in the usual way, proportional to 
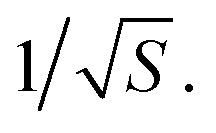
 Hence the error bar for Δ*r*^2^(*t*) computed for a single random walk of length *T* scales as 
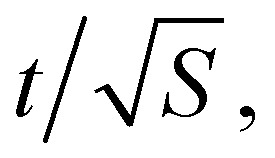
 or *t*^3/2^/*T*^1/2^.

We verify this scaling argument (and determine the prefactor) by explicit calculation, as follows. Consider a three-dimensional random walk of *N* uncorrelated steps {**a**_*i*_}, which consist of components {*x*_*i*_, *y*_*i*_, *z*_*i*_}, each of which are Gaussian distributed with zero mean and unit variance.

The displacement **r**(*n*) after *n* steps is given by 
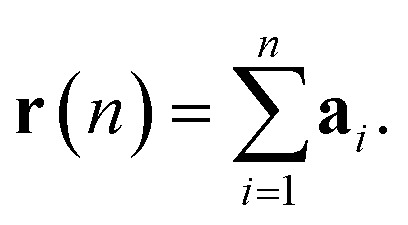
 The mean-square displacement Δ*r*^2^(*n*) after *n* steps, averaged over different starting points *k* in the walk, is15
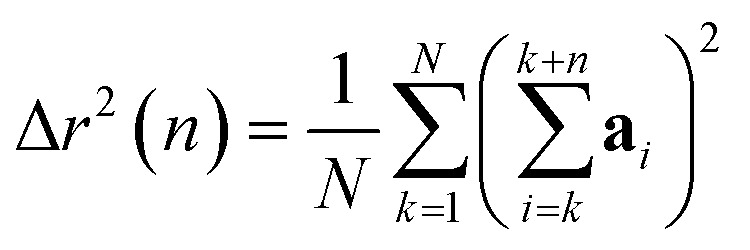


In writing eqn (15), we have averaged over the single random walk {**a**_*i*_}, but have yet not averaged over the ensemble of different random walks. We want to know the error in computing Δ*r*^2^(*n*) this way, *i.e.*, how much Δ*r*^2^(*n*) varies when computed for different random walks. To achieve this, we compute the average 〈Δ*r*^2^(*n*)〉 and variance 〈(Δ*r*^2^(*n*))^2^〉 − 〈Δ*r*^2^(*n*)〉^2^ over the ensemble of random walks.

The walk-averaged mean-square displacement can be written as16
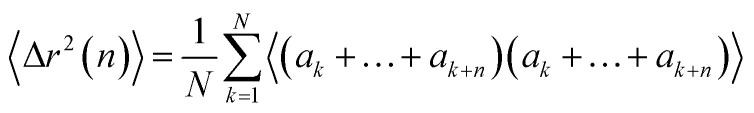


Different steps in the walk are uncorrelated, different components of each step are uncorrelated, and the *x*, *y*, *z* components all have the same distribution, so the average of a term 〈*a*_*k*_·*a*_*j*_〉 is

17 〈*a*_*k*_·*a*_*j*_〉 = 3〈*x*_*j*_*x*_*k*_〉 = 3*δ*_*jk*_

Thus only “diagonal” (*k* = *j*) terms in the expansion of the summand contribute, which results in

18 〈Δ*r*^2^(*n*)〉 = 3*n*

(Equating the walk-averaged mean-square displacement to 6*Dn*, we see that *D* = 1/2 for this choice of step size and statistics.)

Now we evaluate the average over walks of (Δ*r*^2^(*n*))^2^, which can be written as19
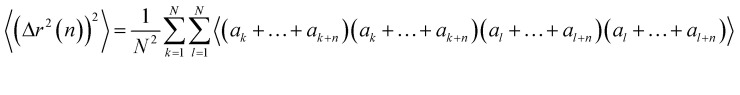


The intervals of steps that appear in the summand, *k*…*k* + *n* and *l*…*l* + *n*, may either be disjoint (if *k* and *l* are far apart compared to *n*) or overlap (if |*k* − *l*| is less than *n*). If the intervals are disjoint, the only surviving contributions to the average are the same as appear in the “factored” average 〈Δ*r*^2^(*n*)〉^2^, so these contributions cancel in computing the variance. So we focus on the contributions in which the intervals overlap, over a number of sites *L* = *n* − |*k* − *l*|.

Examining eqn (19), there are *L* terms in which all four indices are the same, of the form 〈(*a*_*m*_^2^)(*a*_*m*_^2^)〉; and there are *L*^2^ terms in which identical indices occur in pairs, of the form 〈(*a*_*m*_·*a*_*n*_)(*a*_*m*_·*a*_*n*_)〉 and 〈(*a*_*m*_·*a*_*n*_)(*a*_*n*_·*a*_*m*_)〉 with *m* ≠ *n*. These more numerous terms give the leading contribution to the average, so we focus on them. We compute the contribution of each such term as

20 〈(*a*_*m*_·*a*_*n*_)(*a*_*m*_·*a*_*n*_)〉 = 3〈*x*_*m*_^2^〉〈*x*_*n*_^2^〉 = 3

The number of such terms *L* depends on the overlap. As *k*–*j* ranges from −*n* to *n*, *L* ranges from 0 to *n* and back to 0. In eqn (19), we change summation variables in the outer sums to 
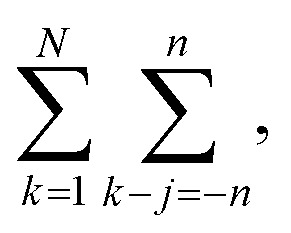
 whereupon we can perform the sum over *k* − *j*. So the total number of contributing terms *T* at each *k* (*i.e.*, as we move along the random-walk trajectory), is21
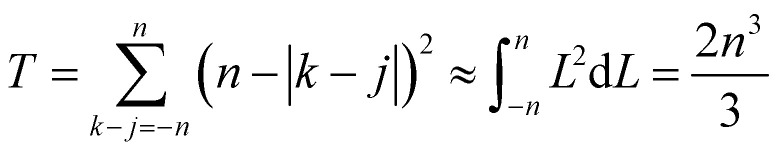


Thus the leading contribution to the variance of the mean-square displacement is22
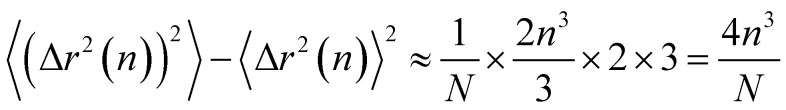
Here the factor of 1/*N* comes from the original sum normalization, the second from the number of contributing terms, the factor of 2 from the two orders of paired indices (〈(*a*_*m*_·*a*_*n*_)(*a*_*m*_·*a*_*n*_)〉 and 〈(*a*·*a*_*n*_)(*a*_*n*_·*a*_*m*_)〉), and the factor of 3 from eqn (20).

Finally, we compute the relative error as23
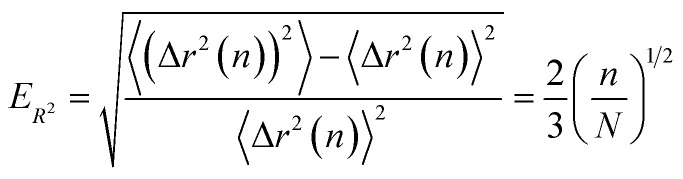


**Fig. 9:**
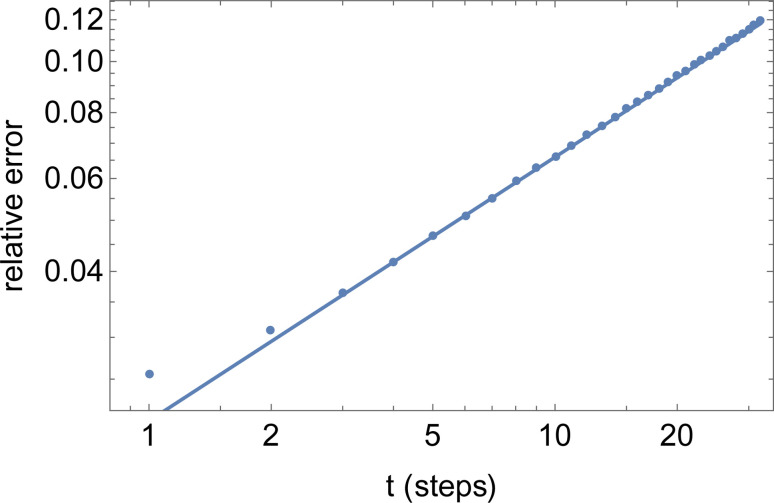
Relative error *versus* number of steps *t* for the mean-square displacement 〈*dr*^2^(*t*)〉 of a single long random walk (here, 1024 steps).

The final result eqn (23) has been verified quantitatively, by generating 4096 instances of a random walk of 1024 steps, and explicitly computing the walk-averaged mean-square displacement and its variance, as a function of the delay *n*. Fig. 9 presents a log–log plot of the resulting relative error (points) *versus* the prediction eqn (23).

## Data availability

The data analysis code and the input trajectory files used in this article are available as an interactive Jupyter notebook at https://github.com/pramudit99/onsager-matrix.git.

## Author contributions

Pramudit Tripathi: methodology, data curation, formal analysis, investigation, software, writing-original draft, validation, visualization. Scott T. Milner: conceptualization, funding acquisition, methodology, project administration, software, supervision, writing-review and editing.

## Conflicts of interest

There are no conflicts to declare.
